# SEM/EDS Evaluation of the Mineral Deposition on a Polymeric Composite Resin of a Toothpaste Containing Biomimetic Zn-Carbonate Hydroxyapatite (microRepair^®^) in Oral Environment: A Randomized Clinical Trial

**DOI:** 10.3390/polym13162740

**Published:** 2021-08-16

**Authors:** Andrea Butera, Maurizio Pascadopoli, Simone Gallo, Marco Lelli, Fabrizio Tarterini, Federico Giglia, Andrea Scribante

**Affiliations:** 1 Unit of Dental Hygiene, Section of Dentistry, Department of Clinical, Surgical, Diagnostic and Pediatric Sciences, University of Pavia, 27100 Pavia, Italy; andrea.butera@unipv.it (A.B.); federico.giglia01@universitadipavia.it (F.G.); 2 Unit of Orthodontics and Pediatric Dentistry, Section of Dentistry, Department of Clinical, Surgical, Diagnostic and Pediatric Sciences, University of Pavia, 27100 Pavia, Italy; 3 Department of Industrial Chemistry “Toso Montanari”, University of Bologna, 40126 Bologna, Italy; lelli.marco1@gmail.com (M.L.); fabrizio.tarterini@unibo.it (F.T.)

**Keywords:** biomimetic, dentistry hydroxyapatite, microrepair, toothpaste, composite resin, polymer composite, mineral deposition, remineralization, SEM, EDS

## Abstract

Toothpastes containing biomimetic hydroxyapatite have been investigated in recent years; the behavior of this material in the oral environment has been evaluated directly on dental enamel showing a marked remineralizing activity. To propose microRepair^®^-based toothpastes (Zn-carbonate hydroxyapatite) for the domiciliary oral hygiene in patients with dental composite restorations, the aim of this study was to evaluate the deposition of Zn-carbonate hydroxyapatite on a polymeric composite resin with Scanning Electron Microscopy/Energy-Dispersive X-ray Spectrometry (SEM/EDS) analysis. Twenty healthy volunteers underwent the bonding of 3 orthodontic buttons on the vestibular surfaces of upper right premolars and first molar. On the surface of the buttons, a ball-shaped mass of composite resin was applied and light-cured. Then, the volunteers were randomly divided into two groups according to the toothpaste used for domiciliary oral hygiene: the Control toothpaste containing stannous fluoride and the Trial toothpaste containing microRepair^®^. The buttons were debonded after 7 days (T1—first premolar), after 15 days (T2—second premolar), and after 30 days (T3—first molar) to undergo the SEM/EDS analysis. The deposition of calcium, phosphorus, and silicon was assessed through EDS analysis and data were submitted to statistical analysis (*p* < 0.05). SEM morphologic evaluation showed a marked deposition of the two toothpastes on the surfaces of the buttons. EDS quantitative analysis showed an increase of calcium, phosphorus, and silicon in both the groups, with a statistically significant difference of calcium deposition at T3 for the Trial group. Therefore, the use of toothpaste containing Zn-carbonate hydroxyapatite could be proposed as a device for domiciliary oral hygiene because the deposition of hydroxyapatite on polymeric composite resin could prevent secondary caries on the margins of restorations.

## 1. Introduction

Dental decay is regarded as one of the most frequent conditions affecting people worldwide. This disease arises from a complex interaction over time occurring between acid-producing bacteria and fermentable carbohydrate from the diet. However, many factors play a crucial role for the development of dental decay, among which are physical, biological, environmental, and behavioral ones. In particular, an insufficient fluoride exposure, an inadequate salivary flow, a high number of cariogenic bacteria, and an improper oral hygiene, all represent important risk factors for the development of caries [1]. 

Presently, restorative therapies are aimed at removing infected dental tissues and at replacing them with composite resins, obtaining proper aesthetic and mechanical results. Nevertheless, an incomplete polymerization of the monomers contained in these materials [2] or an inadequate bonding procedure, also depending on the type of the adhesive system used [3], cause the so-called “microleakage”, that is the formation of a gap between the resin and the dental tissue, thus predisposing to a possible secondary decay. 

Fluoride products have always been used with the aim of promoting the remineralization of teeth. In recent years, an increasing interest was created by remineralizing technologies that have been proposed, among which are the use of calcium-phosphate system, P11-4 peptides, leucine-rich amelogenin peptides, poly (amido amine) dendrimers, and hydroxyapatite [Ca_5_(PO_4_)_3_OH] [4]; in particular, this latter represents one of the most recent remineralization systems which is based on an innovative biomimetic approach aimed to restore the tooth with the same substance constituting its hard tissues [5].

Recent studies in the literature have investigated the efficacy of particulate hydroxyapatite in different clinical situations, such as in preventing caries, periodontitis, and acid erosion, in remineralizing enamel and dentin affected by early carious lesions, and in reducing gingival bleeding and dental hypersensitivity [6,7]. However, most of these studies evaluated the behavior of hydroxyapatite-based toothpastes when applied on the surface of dental enamel [8,9,10,11,12,13], whereas the interactions between this biomimetic material with dental composite resins, in particular at the resin-enamel interface, have not been fully investigated. Only two works dealt with this aspect, evaluating the influence of hydroxyapatite on the shear bond strength of resin composites to dentin [14] and the occurrence of color changes during brushing and coffee staining [15]. 

The properties of composite resins and the type of cavity preparation that predispose to the formation of microleakages, together with host factors, are likely to lead to secondary caries [3]; in particular, the possibility of adequately sealing the tooth-restoration margin with a toothpaste for daily oral hygiene could play a pivotal role for the prevention of secondary caries on composite resin restored teeth [16].

Therefore, the aim of the present study is to test a Zn-carbonate hydroxyapatite-based toothpaste assessing the mineral deposition on the surface of a bulk-fill composite resin (applied on orthodontic buttons subsequently bonded in vivo to the teeth) after a 1-month domiciliary use. Accordingly, the statistical null hypotheses of this study are that there are no significant intergroup and intragroup differences as regards the mineral deposition on the surface of the polymeric composite resin, if exposed to either the toothpaste containing Zn-carbonate hydroxyapatite or a control product.

## 2. Materials and Methods

### 2.1. Trial Design

The study was approved by the Unit Internal Review Board (IRB-2021-0217), and it was registered on clinicaltrials.gov, accessed on 31 June 2021. (registration number: NCT04808557). It was a parallel group, randomized, active controlled, and single-center trial with a 1:1 allocation ratio. 

### 2.2. Participants

20 healthy volunteers leading to the Unit of Dental Hygiene, Section of Dentistry, Department of Clinical, Surgical, Diagnostic and Pediatric Sciences, University of Pavia, Pavia, Italy were enrolled. Recruitment started in March 2021 and the study ended in May 2021. The informed consent was obtained for each participant. 

The inclusion criteria were the following: at least 18 y.o., no current orthodontic treatment during the study, and no use of occlusal splints or retention devices. The exclusion criteria were the presence of teeth vestibular surfaces corrupted or with white spot lesions.

### 2.3. Intervention

**Figure 1:**
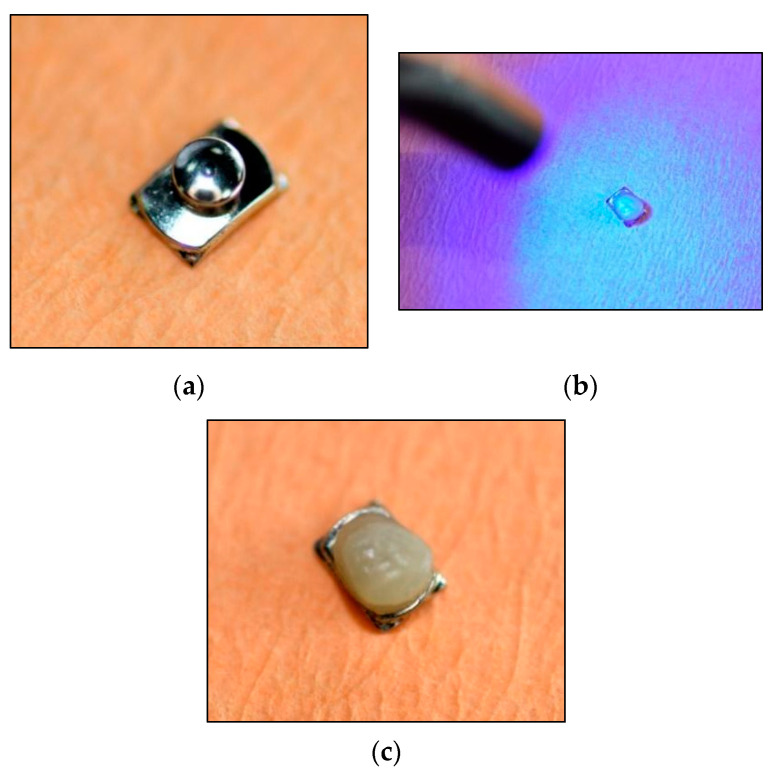
Preparation of the orthodontic button: (**a**) orthodontic button before composite application; (**b**) light curing of the composite applied on the button; (**c**) orthodontic button after the procedure.

A ball-shaped mass of bulk-fill resin composite (Filtek ™ *Supreme* A3B, 3M Unitek, St. Paul, MN, USA) was applied on 60 curved-base lingual buttons (3M Unitek, Monrovia, CA, USA) and light-cured for 20 s with an LED unit (Starlight Pro, Mectron s.p.a., Carasco, Italy) (Figure 1).

Volunteers underwent the bonding procedure of the buttons on the vestibular surfaces of upper right first and second premolars, and upper first molar according to a common protocol for bonding [17]: the vestibular surfaces of the teeth were etched for 30 s with 37% orthophosphoric acid (Gerhò Etchant gel 37%, Gerhò spa, Terlano, Italy); then, after rinsing and drying, a thin layer of Transbond XT Light Cure Adhesive Primer (3M Unitek) was applied and then cured for 10 s with the LED unit (Starlight Pro). At last, Transbond XT Light Cure Adhesive Paste (3M Unitek) was applied on the base of the buttons; the buttons were applied on the vestibular surfaces with a light pressure and the extra paste was removed; curing was performed with the LED unit at 2-3 mm distant from the enamel-button interface for 40 s, 10 s per each surface. 

All participants were instructed to perform correct oral hygiene and had to use a medium-bristled Biorepair manual toothbrush (Coswell S.p.A., Funo di Argelato, Bologna, Italy) for the domiciliary procedures. Volunteers were randomly divided into two groups: in the Trial group, Biorepair Total Protection toothpaste containing microRepair^®^ 200 mg/g (Coswell S.p.A.) was used for home oral hygiene for 30 days twice a day and for 2 min per time, while in the Control Group Sensodyne Repair & Protect toothpaste (GSK Consumer Healthcare S.p.A., Baranzate, Milan, Italy) was used for the same purpose and duration. The compositions of both toothpastes and the composite resin applied on the buttons are shown in Table 1.

After 7 days from the bonding procedure, the buttons on the upper right first premolar were debonded; after 15 days, the buttons on the upper right second premolar were debonded; after 30 days, the remaining buttons were debonded. After the debonding, the buttons were stored in sterile environment and were sent to the Department of Industrial Chemistry “Toso Montanari”, University of Bologna, Bologna, Italy, for the Scanning Electron Microscopy/Energy-Dispersive X-ray Spectrometry (SEM/EDS) analysis.

### 2.4. Outcomes

Each button underwent SEM analysis (SEM EVO 50 EP, Carl Zeiss Inc., Cambridge, UK) for the morphological characterization. Images were taken at 100×, 500× and 2000× magnifications. The manual application of the composite resin on the orthodontic bonding made the morphology of the samples non-homogeneous; consequently, also the deposition of the two toothpastes on the composite resin surface was supposed to happen irregularly. Therefore, only the top of each button was considered for the EDS analysis, as it was believed to be the area most interested by brushing and with the higher mineral deposition. 

EDS elemental analysis was performed with EDAX Inca Energy 350 X-Max50 detector (Oxford Instruments, Abingdon, Oxfordshire, UK). The analyses were conducted at variable pressure, which was used to test the samples without performing a coating procedure of the superficial layer that could alter the surface of the composite resin. For each button, 10 images were taken in random surfaces of the top of the button at 2000× magnification. Elemental mapping was performed for phosphorus, calcium, and silicon. Each image was exported in a JPG file and then processed with Adobe Photoshop CS6 (Adobe Incorporated, Mountain View, CA, USA) to determine the deposition of different elements on the composite resin. “Histogram” tool was used to exploit grayscale percentages: black corresponds to the absence of the element mapped, white, instead, to the maximum amount of deposition. Therefore, the percentages of gray corresponded to the superficial amount of deposition of the considered element. 

### 2.5. Sample Size

**Figure 2:**
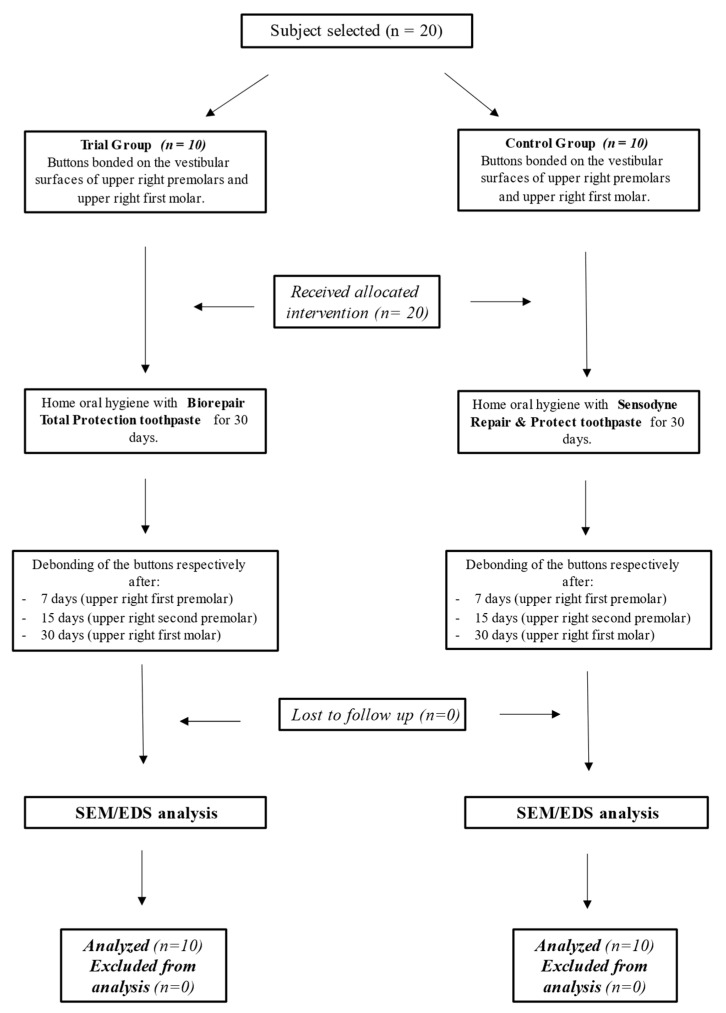
Flow-chart of the study. Legend: SEM, Scanning Electron Microscope; EDS, Energy-Dispersive X-ray Spectrometry.

Concerning the variable “percentage of phosphorus deposition”, an expected mean of 28.19 was hypothesized, with a standard deviation of 1.74. The expected difference between the means was supposed to be 2.36 [12], therefore 10 patients were requested for each group. Accordingly, sample size calculation (Alpha 0.05; Power = 85%) for two independent study groups and a continuous primary endpoint required 20 total participants (50% males and 50% females, mean age 23.5 years old), of which 10 belonging to Control group (50% males and 50% females, mean age 23.1 years old) and 10 belonging to Trial group (50% males and 50% females, mean age 23.9 years old). A total of 20 healthy volunteers were enrolled and all of them agreed to participate and completed the study. Interim analysis and stopping guidelines were not applicable. The flow-chart of the study is shown in Figure 2. 

### 2.6. Sequence Generation

A randomized sequence was generated with a software R (version 3.1.3, R Development Core Team, R Foundation for Statistical Computing, Wien, Austria) using a block randomization table and considering a permuted block randomization with 10 participants for each of the two fixed blocks.

### 2.7. Allocation Concealment

The operator who enrolled participants also achieved the allocation concealment using sequentially numbered, opaque, and sealed envelopes (SNOSE) containing the allocation cards previously prepared. The randomization list generated was held securely in a remote location.

### 2.8. Implementation

The random allocation sequence list was generated by the operator who subsequently performed data analyses. Participants were enrolled by another operator who also assigned them to the respective treatment and did not take part in the subsequent phases of the study.

### 2.9. Blinding

Both the operator that performed the bonding/debonding procedures, the outcome assessor, and the data analyst were blinded during the study. The data analyst and the outcome assessor neither took part in the clinical visits. Patients were not aware of the treatment to which they were subjected because it was possible to conceal the two toothpastes, except for the taste. However, this latter factor was considered irrelevant since the study was not conceived as a split-mouth design and the outcomes assessed were not subjective. 

### 2.10. Statistical Methods

Data analysis was conducted with R software (R version 3.1.3, R Development Core Team, R Foundation for Statistical Computing, Wien, Austria). For each variable, descriptive statistics were calculated for both the Trial and the Control groups. Data included mean, standard deviation, minimum, median, and maximum percentage values for each ion tested. Kolmogorov and Smirnov test was applied to assess normality of distributions. Subsequently, repeated measures ANOVA test was calculated, followed by Tukey’s test for post-hoc analysis. Significance for all statistical tests was predetermined at *p* < 0.05. 

## 3. Results

### 3.1. Morphological Characterization

**Figure 3:**
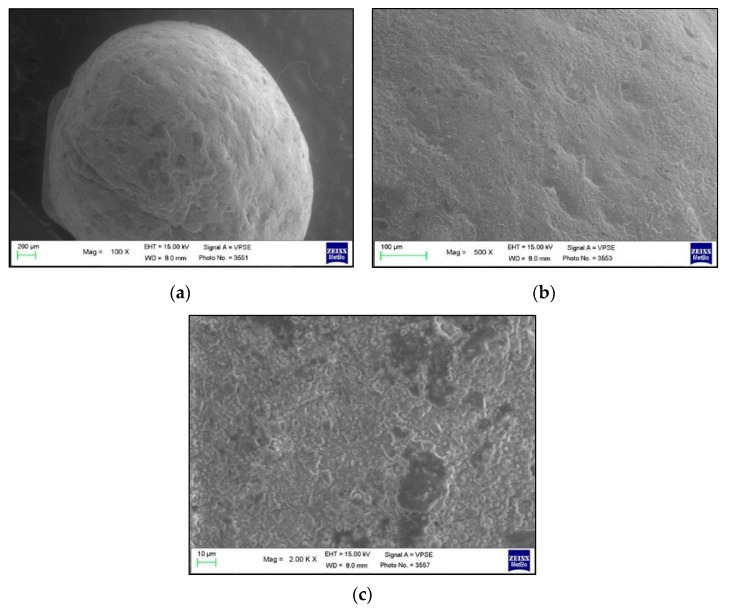
SEM images of the orthodontic button at baseline (T0, before bonding) at different magnifications: (**a**) 100×; (**b**) 500× and (**c**) 2000×.

In Figure 3, an example of an orthodontic button covered with the resin composite is shown at baseline (T0), before the bonding procedure to the tooth. The images, at 100×, 500× and 2000× magnifications show an irregular surface of the composite resin, with particles of different sizes, randomly assembled, but with a similar morphology. 

**Figure 4:**
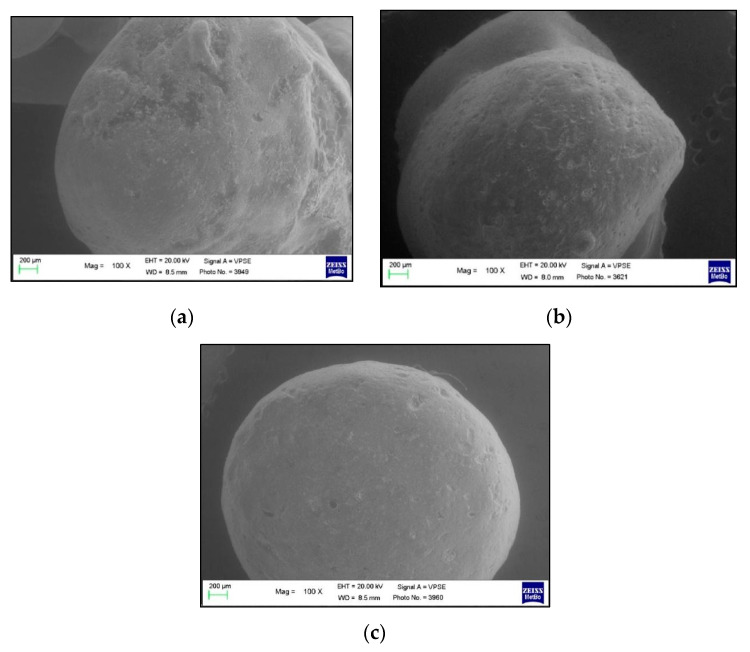
100× magnification of a button belonging to the Control group at (**a**) T1 (7 days); (**b**) T2 (15 days); and (**c**) T3 (30 days).

**Figure 5:**
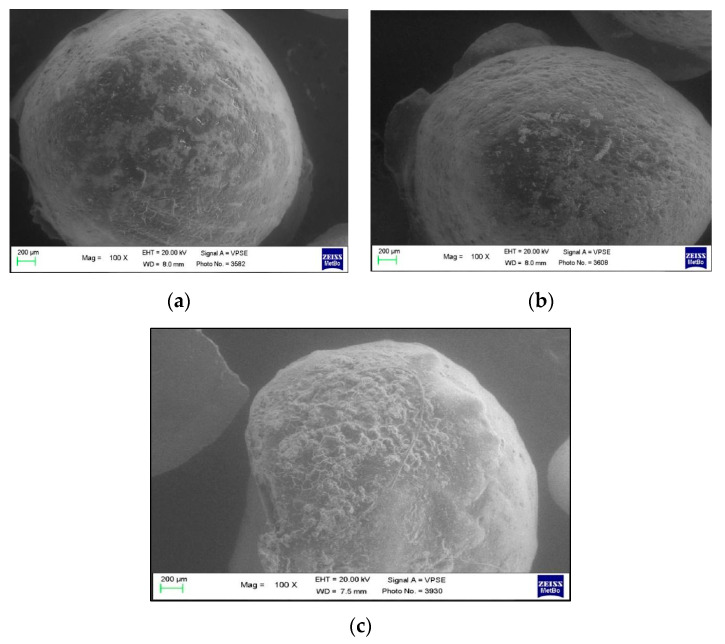
100× magnification of a button belonging to Trial group at (**a**) T1 (7 days); (**b**) T2 (15 days); and (**c**) T3 (30 days).

The use of both the Control toothpaste (Figure 4) and the Zn-carbonate hydroxyapatite-based one (Figure 5) determined the formation of dark gray spots on the surface of the buttons. The spots are morphologically different from the composite resin and are identifiable; in fact, the button appears smoother and with fewer irregularities. 

**Figure 6:**
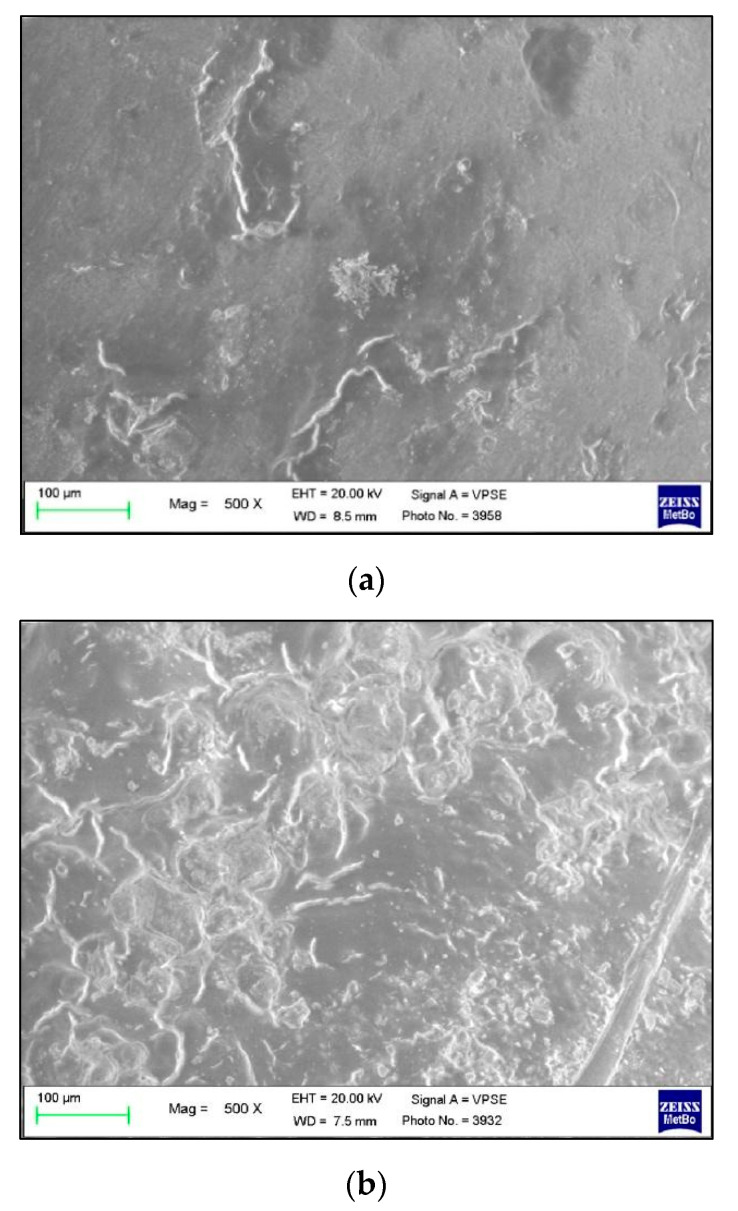
500× magnification of buttons at T3 belonging to (**a**) Control group and (**b**) Trial group.

The images show that the dark gray spots increase from T1 to T3, probably due to the deposition of mineral salts and inorganic phases contained in the two toothpastes. The Trial group showed the presence of more spots if compared to the Control group. For both the groups, the presence of the spots resulted inhomogeneous (Figure 6).

### 3.2. Elemental Analysis

**Figure 7:**
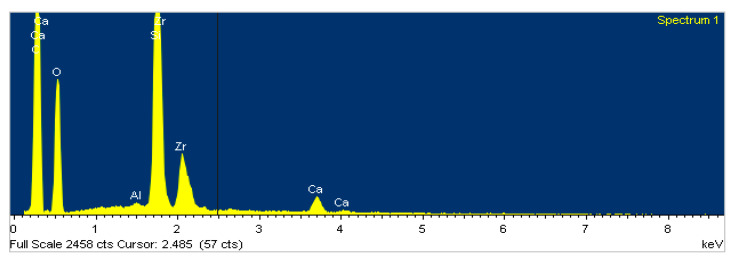
Elemental spectrum of composite resin surface of the button before bonding (Untreated –T0).

EDS analysis was conducted before the bonding inside the mouth (baseline, T0) on a representative polymeric composite mass, to assess the composition of the resin not exposed to the toothpastes and to consider these as blank values. On the basal resin surface, the following elements were found: carbon, oxygen, aluminum, silicon, zirconium, calcium (Figure 7).

All the polymeric composite masses at T1, T2, and T3 were respectively submitted for EDS analysis. In relation to the composition of the two toothpastes, the major components found are hydrated silica for both groups, pentasodium triphosphate for the Control group and microRepair^®^ (Zn-carbonate hydroxyapatite) for the Trial group. Subsequently, the percentages of calcium, phosphorous, and silicon deposition on the surfaces of the composite resin of the buttons were quantitatively determined. 

### 3.3. Percentage of Calcium Deposition (Ca%)

The descriptive statistics for the relative percentages of calcium ions are shown in Table 2. 

**Figure 8:**
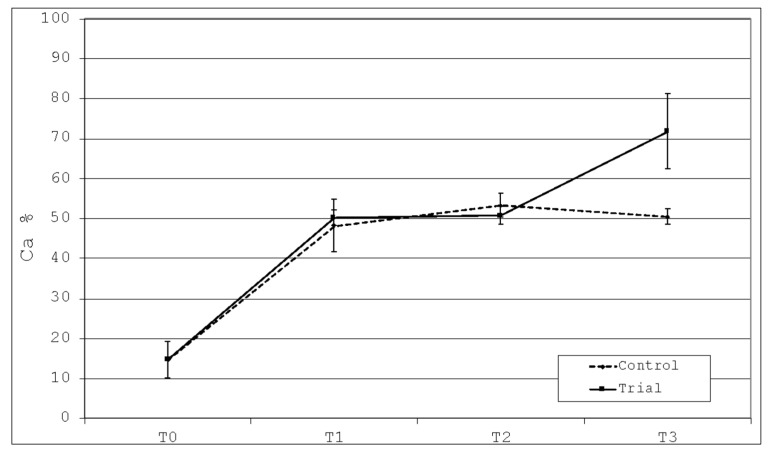
Graphical evaluation of calcium deposition among the time frames of the study.

There is a statistically significant difference between T0 and T1 for both Control and Trial groups (*p* < 0.05). In the Control groups, no statistically significant difference was found between T1, T2 and T3 (*p* > 0.05). In the Trial group, no statistically significant difference was found between T1 and T2 (*p* > 0.05), whereas there was a significant increase between T2 and T3 (*p* < 0.05) with higher values with respect to the Control group at T3 (Figure 8). 

### 3.4. Percentage of Phosphorus Deposition (P%)

The descriptive statistics for the relative percentages of phosphorus ions are shown in Table 3. 

**Figure 9:**
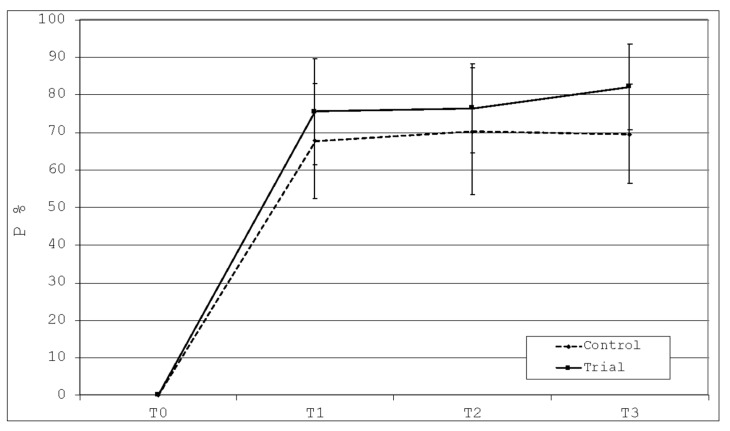
Graphical evaluation of phosphorus deposition among the time frames of the study.

After the application of the two toothpastes, there was a statistically significant increase in the percentages of deposition of phosphorus in both the groups at T1 (*p* < 0.05). Conversely, no statistically significant intragroup differences occurred at T1, T2, and T3 (*p* > 0.05), despite the Trial group showed higher values if compared to the Control one (Figure 9). 

### 3.5. Percentage of Silicon Deposition (Si%)

The descriptive statistics for the relative percentages of silicon ions are shown in Table 4. 

**Figure 10:**
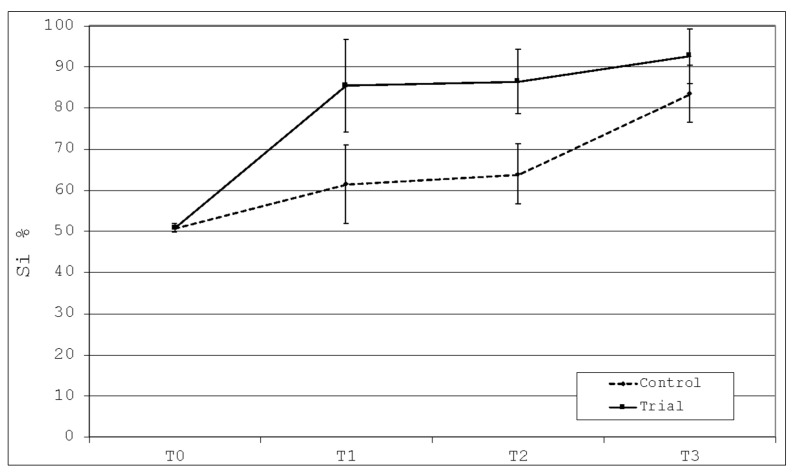
Graphical evaluation of silicon deposition among the time frames of the study.

There is a statistically significant increase of silicon deposition at T1 in both groups (*p* < 0.05). No significant intragroup difference was found between T1 and T2 in the Control group and between T1, T2 e T3 in the Trial group (*p* > 0.05). A significant intergroup difference was found at T1 and T2 (*p* < 0.05). In the Control group, a significant increase was found between T2 and T3 (*p* < 0.05) (Figure 10). 

## 4. Discussion

The phenomenon of tooth wear represents a clinical challenge which dental practitioners must face during everyday dental practice. This condition encompasses different clinical entities affecting enamel, i.e., abrasion, attrition, and erosion. In particular, this latter is by far the most frequent cause of tooth wear, and it consists of the dissolution of the dental hydroxyapatite following an exposition to extrinsic or intrinsic acids, not deriving from the bacterial metabolism [18]. Many efforts have been done to contrast the process of tooth demineralization, especially with the use of products for the domiciliary oral hygiene containing specific substances capable of remineralizing and/or even repairing the dental surface. In recent years, hydroxyapatite has been investigated for its various fields of application, among which is dentistry, where it has been synthesized for the incorporation in toothpastes for oral hygiene, expressing a remineralizing activity both on enamel and dentine [5].

Many studies have been conducted both in vitro and in vivo. Among the former, Vyavhare et al. [19] and Thimmaiah et al. [20] assessed the remineralizing efficacy of biomimetic hydroxyapatite after artificial demineralization, while Nasution and Basri [21] found no efficacy, probably because of the methodology used for the study: in fact, the specimens were immersed in different solutions and then consequently centrifuged, instead of evaluating the effects of brushing during a precisely time frame. 

Among the in vivo studies, instead, Najibfard et al. [22] assessed for the first time the in vivo remineralizing activity of hydroxyapatite; they used enamel blocks obtained from sections of sound extracted third molars, then carious lesions were artificially produced. The enamel blocks were covered with a polyester gauze and bonded to customized orthodontic brackets. Four different appliances were bonded for a 28 days-period each, to evaluate the behavior of two toothpastes containing 5% and 10% hydroxyapatite and a NaF toothpaste. The results highlighted an equal remineralizing activity of the former toothpastes if compared to the latter one. 

The study of Lelli et al. [11] evaluated the effect exerted by a toothpaste containing Zn-CHA structured microcrystal on the enamel, with respect to a potassium nitrate/sodium fluoride toothpaste (active control) and a fluoride toothpaste (negative control). After extraction, teeth exposed to the toothpastes underwent morphological and physical-chemical superficial characterizations. The results of the study showed that the use of the Zn-CHA crystal toothpaste caused a remineralization/repair of the enamel structure with an evident deposition of a mineral layer, with respect to the other toothpastes not altering the enamel surface. A similar protocol was exploited by Bossù et al. [9] in a pediatric sample to assess the remineralizing efficacy on primary teeth of different products, among which is a toothpaste containing biomimetic hydroxyapatite. The results showed a typical morphological characterization and significantly lower roughness values in the teeth brushed with Biorepair toothpaste. This occurrence was assessed also in an observational multicentric study [23] in which patients perceived a subjectively increase in smoothness after brushing twice a day for 28 days with biomimetic hydroxyapatite containing toothpaste; however, it was an open label trial, therefore the results could not be reliable at all. Amaechi et al. [24] compared a fluoridated and a hydroxyapatite-based toothpastes in the remineralization of deciduous teeth finding no significant differences and suggesting the latter as for its dose-dependent non-toxicity. Badiee and colleagues [25], instead, conducted a clinical trial in which orthodontic patients, enrolled after debonding, were asked to use a fluoride and a hydroxyapatite containing toothpastes for 6 months; the latter toothpaste showed a greater entity of remineralization of the vestibular surfaces of teeth where brackets were bonded. Schlagenhauf et al. [26] found no significant difference in caries inhibition during fixed orthodontic treatment after 6 months of oral hygiene performed with a non-fluoridated microcrystalline hydroxyapatite dentifrice in respect to a fluoridated one; however, it should be noted that the participants were young highly caries-susceptible orthodontic patients and belonging to the same age range.

Despite the properties of hydroxyapatite have been particularly examined as regards its effects towards the natural surface of the tooth, the interaction between this substance and the interface between enamel and restorative materials has not been investigated as well. However, the frequent gap formation between the polymeric resin and the dental tissue called “microleakage” is quite frequent and predisposes to the risk of secondary decay [2]. Based on this consideration, the goal of the present report has been that of evaluating the deposition of biomimetic hydroxyapatite on a polymeric restorative composite resin. The null hypotheses of the study have been partially rejected. In fact, in respect to the untreated button, both the toothpastes contributed to release calcium, phosphate, and silicon on the surfaces of the composite resin. Specifically, calcium deposition has increased equally in both the groups until T2. At T3, there was a statistically significant increase in the calcium absorption in the Trial group in respect to the Control group. Phosphorus deposition has increased in both the groups until T3, with a non-significant higher deposition in the Trial group. At last, for silicon deposition statistically significant differences were found at T1 and T2, as the percentage of silicon increases in both groups, but faster and more markedly in the Trial group. At T3, there is still an increase of the percentage between the two groups, but with no statistically significant difference. Therefore, as phosphorus and calcium are the main constituents of hydroxyapatite, we can assess that there was a deposition of these ions on the surface of the composite resin after 1 month brushing with Biorepair toothpaste. The Control group showed deposition as well, but lower as regards calcium and slower for the silicon. 

In addition to daily oral hygiene, another approach to the issue of preventing secondary caries could be from the incorporation of particles of hydroxyapatite into restorative dental materials, as was done for glass-ionomer cements [27] determining an improvement on shear bond strength and flexural strength values, and for composite resin [28] leading to calcium and phosphate release under acidic circumstances, not without alterations of physical-chemical properties of the restorative material. Particles of hydroxyapatite have been added to resin-modified glass-ionomer cements, resulting in a reduction of the microleakage if compared to conventional glass-ionomer cements [16]. Considering that the mechanical properties of dental restorative materials may be altered after the incorporation of hydroxyapatite, further studies are required to obtain an improvement of their mechanical properties. Moreover, another approach to the issue could be the exploitation of the antibacterial effect exerted by ozone; in fact, the combination of ozone with a gel containing hydroxyapatite was proven to be effective in remineralizing initial approximal enamel lesions in a 2-in year follow up trial in respect to gel alone and ozone alone [29].

An important consideration which deserves to be done is the comparison of biomimetic hydroxyapatite with respect to fluoride. Mouthwashes and toothpastes containing fluoride ions are among the most popular products for oral hygiene. Fluoride ions have the capability of interacting with dental hydroxyapatite crystals, thus forming the less insoluble fluoridated hydroxyapatite or fluorapatite, which are more resistant to the acid attack [30]. However, the efficacy of fluoride toothpastes is limited to a partial substitution of the hydroxyl groups with fluoride ions in natural hydroxyapatite, with no deposition of an additional mineral content [31]. Conversely, as demonstrated in the present study, biomimetic hydroxyapatite even leads to a mineral deposition, thus forming a real coating on enamel and dental surfaces [11]. Additionally, the use of fluoride-based products may be linked to a risk of toxicity in the case of high dosage intake, respectively consisting of fluorosis in children and bone diseases in the elderly. The European Food Safety Authority (EFSA) suggests that the maximum level of fluoride content for oral care products, including toothpastes, is 1500 mg/Kg. As well, the maximum fluoride intake should be 0.1 mg fluoride/kg/day in children aged 1–8 years (Lelli 2013). Based on this consideration, in addition to the different remineralizing effect, to lower the risk of toxicity linked to fluoride might suggest the safer use of biomimetic hydroxyapatite in young children [9,24,26]. 

One of the limitations of this study is that saliva-related factors of the participants were not considered, also if the availability of calcium and phosphate ions does not seem to influence the deposition of hydroxyapatite, as it happens for fluoride toothpastes [24].

Another limit regards the composite resin used. In fact, only a uniform surface of composite resin was tested for the evaluation of the mineral deposition and samples with an enamel/resin interface would be suitable for further in vitro/in vivo investigations. Moreover, only one type of bulk-filled composite resin was tested, while it would be interesting to evaluate other types of composite resins. 

Moreover, the percentages of hydroxyapatite contained in toothpastes change, so testing different products would help in assessing the best formulation. 

At last, further evaluations should be performed considering the brushing with electric toothbrushes. 

Therefore, future perspectives should consider improving dental materials incorporating hydroxyapatite and to suggest the use of a toothpaste containing biomimetic hydroxyapatite for domiciliary oral hygiene as a simultaneous approach for contrasting microleakage caries. Additionally supplementary physical-chemical tests should be encouraged as a help for the evaluation of the amount of hydroxyapatite deposition. 

## 5. Conclusions

The present study demonstrated that there was a deposition of calcium, phosphorus and silicon ions on the surfaces of bulk-filled polymeric composite resins in oral environment after one month of daily oral hygiene with a toothpaste containing microRepair^®^ (Zn-carbonate hydroxyapatite). The results showed a higher deposition of calcium in respect to the Control group after 1 month of brushing; the phosphorus deposited in the same way comparing the two groups, while silicon deposited faster in the Trial one, despite in the Control group the quantity of silicon did not significantly differ after 1 month if compared to the experimental group. 

Therefore, the use of a toothpaste containing microRepair^®^ could be a recommended device for home oral hygiene in patients with composite resin restorations, because in addition to the proven remineralizing activity on dental enamel, an effective deposition on the surface of the polymeric composite resin was assessed and a likely sealant efficacy on the microleakage could be exerted.

## Figures and Tables

**Table 1:** Compositions of the resin composite and the toothpastes used for the study.

Material	Manufacturer	Composition
Filtek ™ *Supreme* A3B	3M Unitek (St Paul, MN, USA)	Bis-GMA, UDMA, TEGDMA, Bis-EMA, non-agglomerated silica, non-agglomerated and agglomerated zirconia, and aggregated particles of zirconia/silica.
Biorepair Total Protection	Coswell, S.p.A. (Funo di Argelato, Bologna, Italy)	Aqua, microRepair^®^ 200 mg/g (zinc-substituted-carbonate-hydroxyapatite crystals) glycerin, hydrated silica, sorbitol, silica, aroma, cellulose gum, sodium myristoyl sarcosinate, sodium methyl cocoyl taurate, citric acid, tetrapotassium pyrophosphate, zinc PCA, sodium saccharin, phenoxyethanol, benzyl alcohol, sodium benzoate.
Sensodyne Repair & Protect	GSK Consumer Healthcare S.p.A. (Baranzate, Milan, Italy)	Stannous fluoride, glycerin, PEG-8, hydrated silica, pentasodium triphosphate, sodium lauryl sulfate, flavor, titanium dioxide, polyacrylic acid, cocamidopropyl betaine, sodium saccharin.

**Legend**: bis-GMA, bisphenol A diglycidyl ether dimethacrylate; UDMA, urethane dimethacrylate; TEGDMA, triethylene glycol dimethacrylate; bis-EMA, bisphenol A diglycidyl methacrylate ethoxylated; PCA, Pyrrolidone Carboxylic Acid; PEG, polyethilen glycol.

**Table 2:** Percentages of calcium among the time frames of the study.

Ca%	Mean	St Dev	Min	Mdn	Max	Significance *
Untreated T0	14.67	7.50	5.41	14.39	23.36	A
Control T1	48.18	10.48	21.52	48.68	60.44	B
Control T2	53.26	4.78	46.10	52.75	61.32	B
Control T3	50.38	3.09	47.25	49.75	58.12	B
Trial T1	50.15	3.08	46.91	49.75	55.92	B
Trial T2	50.65	3.53	45.25	50.06	57.54	B
Trial T3	71.68	15.31	51.70	70.25	92.20	C

* different letters (A or B) show significant intragroup/intergroup differences (*p* < 0.05). Groups with the same letters are not significantly different (*p* > 0.05).

**Table 3:** Percentages of phosphorus among the time frames of the study.

P%	Mean	St Dev	Min	Mdn	Max	Significance *
Untreated T0	0.00	0.00	0.00	0.00	0.00	A
Control T1	67.74	24.72	29.76	60.81	98.72	B
Control T2	70.21	27.20	0.00	75.97	96.70	B
Control T3	69.44	21.21	43.38	66.88	97.35	B
Trial T1	75.39	22.94	41.41	71.96	99.93	B
Trial T2	76.34	19.26	54.27	74.07	99.50	B
Trial T3	82.06	18.40	52.45	92.83	97.70	B

*: different letters (A or B) show significant intragroup/intergroup differences (*p* < 0.05). Groups with the same letters are not significantly different (*p* > 0.05).

**Table 4:** Percentages of silicon among the time frames of the study.

Si%	Mean	St Dev	Min	Mdn	Max	Significance *
Untreated T0	50.71	1.68	47.67	50.78	53.19	A
Control T1	61.42	15.36	27.21	67.31	74.10	B
Control T2	63.83	11.71	48.03	65.65	82.14	B
Control T3	83.36	11.22	64.37	86.31	95.70	C
Trial T1	85.32	18.02	55.54	96.88	99.77	C
Trial T2	86.33	12.79	62.04	89.48	99.72	C
Trial T3	92.52	10.86	70.88	98.21	99.99	C

* different letters (A or B) show significant intragroup/intergroup differences (*p* < 0.05). Groups with the same letters are not significantly different (*p* > 0.05).

## Data Availability

Data are available upon reasonable request to the Corresponding Authors.

## References

[1] Selwitz R.H., Ismail A.I., Pitts N.B. (2007). Dental caries. Lancet.

[2] Colombo M., Gallo S., Poggio C., Ricaldone V., Arciola C.R., Scribante A. (2020). New Resin-Based Bulk-Fill Composites: In vitro Evaluation of Micro-Hardness and Depth of Cure as Infection Risk Indexes. Materials.

[3] Nedeljkovic I., Teughels W., De Munck J., Van Meerbeek B., Van Landuyt K.L. (2015). Is secondary caries with composites a material-based problem?. Dent Mater..

[4] Philip N. (2019). State of the Art Enamel Remineralization Systems: The Next Frontier in Caries Management. Caries Res..

[5] Roveri N., Iafisco M. (2010). Evolving Application of Biomimetic Nanostructured Hydroxyapatite. Nanotechnol. Sci. Appl..

[6] Enax J., Fabritius H.O., Fabritius-Vilpoux K., Amaechi B.T., Meyer F. (2019). Modes of action and clinical efficacy of particulate hydroxyapatite in preventive oral health care—State of the art. Open Dent. J..

[7] Scribante A., Poggio C., Gallo S., Riva P., Cuocci A., Carbone M., Arciola C.R., Colombo M. (2020). In Vitro Re-Hardening of Bleached Enamel Using Mineralizing Pastes: Toward Preventing Bacterial Colonization. Materials.

[8] Scribante A., Dermenaki Farahani M.R., Marino G., Matera C., Rodriguez Y., Baena r., Lanteri v., Butera a. (2020). Biomimetic Effect of Nano-Hydroxyapatite in Demineralized Enamel before Orthodontic Bonding of Brackets and Attachments: Visual, Adhesion Strength, and Hardness in In Vitro Tests. Biomed. Res. Int..

[9] Bossù M., Saccucci M., Salucci A., Di Giorgio G., Bruni E., Uccelletti D., Sarto M.S., Familiari G., Relucenti M., Polimeni A. (2019). Enamel remineralization and repair results of Biomimetic Hydroxyapatite toothpaste on deciduous teeth: An effective option to fluoride toothpaste. J. Nanobiotechnol..

[10] Kilic M., Gurbuz T. (2021). Evaluation of the effects of different remineralisation agents on initial enamel lesions by scanning electron microscope and energy-distributed X-ray analysis. Int. J. Clin. Pract..

[11] Lelli M., Putignano A., Marchetti M., Foltran I., Mangani F., Procaccini M., Roveri N., Orsini G. (2014). Remineralization and repair of enamel surface by biomimetic Zn-carbonate hydroxyapatite containing toothpaste: A comparative in vivo study. Front. Physiol..

[12] Gjorgievska E.S., Nicholson J.W., Slipper I.J., Stevanovic M.M. (2013). Remineralization of demineralized enamel by toothpastes: A scanning electron microscopy, energy dispersive X-ray analysis, and three-dimensional stereo-micrographic study. Microsc. Microanal..

[13] Kulal R., Jayanti I., Sambashivaiah S., Bilchodmath S. (2016). An In-vitro Comparison of Nano Hydroxyapatite, Novamin and Proargin Desensitizing Toothpastes—A SEM Study. J. Clin. Diagn. Res..

[14] Gupta T., Nagaraja S., Mathew S., Narayana I.H., Madhu K.S., Dinesh K. (2017). Effect of Desensitization Using Bioactive Glass, Hydroxyapatite, and Diode Laser on the Shear Bond Strength of Resin Composites Measured at Different Time Intervals: An In vitro Study. Contemp. Clin. Dent..

[15] Turgut S., Kılınç H., Ulusoy K.U., Bagis B. (2018). The Effect of Desensitizing Toothpastes and Coffee Staining on the Optical Properties of Natural Teeth and Microhybrid Resin Composites: An In-Vitro Study. Biomed. Res. Int..

[16] Sharafeddin F., Feizi N. (2017). Evaluation of the effect of adding micro-hydroxyapatite and nano-hydroxyapatite on the microleakage of conventional and resin-modified Glass-ionomer Cl V restorations. J. Clin. Exp. Dent..

[17] Scribante A., Gallo S., Turcato B., Trovati F., Gandini P., Sfondrini M.F. (2020). Fear of the Relapse: Effect of Composite Type on Adhesion Efficacy of Upper and Lower Orthodontic Fixed Retainers: In Vitro Investigation and Randomized Clinical Trial. Polymers.

[18] Shellis R.P., Addy M. (2014). The interactions between attrition, abrasion and erosion in tooth wear. Monogr. Oral. Sci..

[19] Vyavhare S., Sharma D.S., Kulkarni V.K. (2015). Effect of three different pastes on remineralization of initial enamel lesion: An in vitro study. J. Clin. Pediatr. Dent..

[20] Thimmaiah C., Shetty P., Shetty S.B., Natarajan S., Thomas N.A. (2019). Comparative analysis of the remineralization potential of CPP-ACP with Fluoride, Tri-Calcium Phosphate and Nano Hydroxyapatite using SEM/EDX—An in vitr ostudy. J. Clin. Exp. Dent..

[21] Nasution A.I., Basri A.B.A. (2017). Comparative scanning electron microscopy/energy-dispersive X-ray study of nano-hydroxyapatite toothpaste in correlation of remineralization. Int. J. Contemp. Dent. Med. Rev..

[22] Najibfard K., Ramalingam K., Chedjieu I., Amaechi B.T. (2011). Remineralization of early caries by a nano-hydroxyapatite dentifrice. J. Clin. Dent..

[23] Steinert S., Zwanzig K., Doenges H., Kuchenbecker J., Meyer F., Enax J. (2020). Daily Application of a Toothpaste with Biomimetic Hydroxyapatite and Its Subjective Impact on Dentin Hypersensitivity, Tooth Smoothness, Tooth Whitening, Gum Bleeding, and Feeling of Freshness. Biomimetics.

[24] Amaechi B.T., AbdulAzees P.A., Alshareif D.O., Shehata M.A., Lima P.P.C.S., Abdollahi A., Kalkhorani P.S., Evans V. (2019). Comparative efficacy of a hydroxyapatite and a fluoride toothpaste for prevention and remineralization of dental caries in children. BDJ Open..

[25] Badiee M., Jafari N., Fatemi S., Ameli N., Kasraei S., Ebadifar A. (2020). Comparison of the effects of toothpastes containing nanohydroxyapatite and fluoride on white spot lesions in orthodontic patients: A randomized clinical trial. Dent. Res. J..

[26] Schlagenhauf U., Kunzelmann K.H., Hannig C., May T.W., Hösl H., Gratza M., Viergutz G., Nazet M., Schamberger S., Proff P. (2019). Impact of a non-fluoridated microcrystalline hydroxyapatite dentifrice on enamel caries progression in highly caries-susceptible orthodontic patients: A randomized, controlled 6-month trial. J. Investig. Clin. Dent..

[27] Kheur M., Kantharia N., Lakha T., Kheur S., Al-Haj Husain N., Özcan M. (2020). Evaluation of mechanical and adhesion properties of glass ionomer cement incorporating nano-sized hydroxyapatite particles. Odontology.

[28] Jardim R.N., Rocha A.A., Rossi A.M., de Almeida Neves A., Portela M.B., Lopes R.T., Pires Dos Santos T.M., Xing Y., Moreira da Silva E. (2020). Fabrication and characterization of remineralizing dental composites containing hydroxyapatite nanoparticles. J. Mech. Behav. Biomed. Mater..

[29] Grocholewicz K., Matkowska-Cichocka G., Makowiecki P., Droździk A., Ey-Chmielewska H., Dziewulska A., Tomasik M., Trybek G., Janiszewska-Olszowska J. (2020). Effect of nano-hydroxyapatite and ozone on approximal initial caries: A randomized clinical trial. Sci. Rep..

[30] Lelli M., Marchisio O., Foltran I., Genovesi A., Montebugnoli G., Marcaccio M., Covani U., Roveri N. (2013). Different corrosive effects on hydroxyapatite nanocrystals and amine fluoride-based mouthwashes on dental titanium brackets: A comparative in vitro study. Int. J. Nanomed..

[31] Orsini G., Procaccini M., Manzoli M., Giuliodori F., Lorenzini A., Putignanol A. (2010). A double-blind randomized-controlled trial comparing the desensitizing efficacy of a new dentifrice containing carbonate/ hydroxyapatite nanocrystals and a sodium fluoride/potassium nitrate dentifrice. J. Clin. Periodontol..

